# Machine Learning and Regression Analysis to Model the Length of Hospital Stay in Patients with Femur Fracture

**DOI:** 10.3390/bioengineering9040172

**Published:** 2022-04-14

**Authors:** Carlo Ricciardi, Alfonso Maria Ponsiglione, Arianna Scala, Anna Borrelli, Mario Misasi, Gaetano Romano, Giuseppe Russo, Maria Triassi, Giovanni Improta

**Affiliations:** 1 Department of Electrical Engineering and Information Technology, University of Naples “Federico II”, 80125 Naples, Italy; carlo.ricciardi@unina.it; 2 Department of Public Health, University Hospital of Naples “Federico II”, 80131 Naples, Italy; ariannascala7@gmail.com (A.S.); triassi@unina.it (M.T.); ing.improta@gmail.com (G.I.); 3 Health Department, University Hospital of Salerno “San Giovanni di Dio e Ruggi d′Aragona”, 84126 Salerno, Italy; acquarama@libero.it; 4 Department of the Orthopaedics, National Hospital (A.O.R.N.) Antonio Cardarelli, 80131 Naples, Italy; mario.misasi@aocardarelli.it (M.M.); gaetano.romano@aocardarelli.it (G.R.); 5 National Hospital (A.O.R.N.) Antonio Cardarelli, 80131 Naples, Italy; ariete_gr@libero.it; 6 Interdepartmental Center for Research in Healthcare, Management and Innovation in Healthcare (CIRMIS), University of Study of Naples “Federico II”, 80131 Naples, Italy

**Keywords:** machine learning, multiple linear regression, clinical pathway, orthopaedic

## Abstract

Fractures of the femur are a frequent problem in elderly people, and it has been demonstrated that treating them with a diagnostic–therapeutic–assistance path within 48 h of admission to the hospital reduces complications and shortens the length of the hospital stay (LOS). In this paper, the preoperative data of 1082 patients were used to further extend the previous research and to generate several models that are capable of predicting the overall LOS: First, the LOS, measured in days, was predicted through a regression analysis; then, it was grouped by weeks and was predicted with a classification analysis. The KNIME analytics platform was applied to divide the dataset for a hold-out cross-validation, perform a multiple linear regression and implement machine learning algorithms. The best coefficient of determination (R^2^) was achieved by the support vector machine (R^2^ = 0.617), while the mean absolute error was similar for all the algorithms, ranging between 2.00 and 2.11 days. With regard to the classification analysis, all the algorithms surpassed 80% accuracy, and the most accurate algorithm was the radial basis function network, at 83.5%. The use of these techniques could be a valuable support tool for doctors to better manage orthopaedic departments and all their resources, which would reduce both waste and costs in the context of healthcare.

## 1. Introduction

Fractures of the neck of the femur are very frequent in elderly people (over 65 years of age); the risk for this type of fracture seems to be higher for women than for men [1]. The main causes are linked to falls, or even to apparently insignificant traumas and chronic bone diseases, such as osteoporosis [2]. The international guidelines state that the best treatment for fracture of the neck of the femur is surgery [3]. In particular, the scientific evidence shows that surgery within 48 h of admission is an effective treatment that can significantly reduce complications in the short, medium and long terms [4,5]. For this reason, the A. Cardarelli Hospital has introduced the diagnostic–therapeutic–assistance path (DTAP), which is a clinical pathway that involves a complex intervention for the organization of care processes for a well-defined group of patients over a well-defined period. It is an organizational model that is characterized by the appropriateness of the interventions, by the integration of the skills of the various professional figures and by the creation of the continuity of care between the hospital and the territory [6]. DTAPs support hospitals in the care quality management, thereby promoting organized and efficient patient care [7], and reducing complications, the length of the hospital stay (LOS) and the medical costs [8]. Previous studies have also proven that their implementation, combined with management approaches, such as Lean and Six Sigma, reduces the variability in clinical practice and improves outcomes [9,10,11,12]. In the specific case of the A. Cardarelli Hospital, the DTAP describes the approaches, organizational procedures and processes that are mandatory for all hospital workers, with the aim of speeding up the surgical process for patients with femoral neck fractures. This clinical pathway includes three phases: (1) Initial patient care after arrival in the emergency room; (2) Perioperative management; and (3) The predischarge period. The implementation of the DTAP, in this case, significantly improved the management of patients with femoral neck fractures; indeed, Six Sigma studies by Improta et al. and Ricciardi et al. show that the implementation of a DTAP led to a significant reduction in the preoperative LOS and in the overall LOS, respectively, at the A. Cardarelli Hospital [13,14].

Mathematical modelling has been used in the healthcare sector for various purposes: Tesfahun et al. developed a model that can predict the rate of the production of medical waste to optimize the management processes of this waste [15] and to predict the spread of viruses and bacteria [16,17]. In this situation, multiple linear regression was used to assess which factors influence the LOS in patients with dengue haemorrhagic fever [18]. Similarly, Liu et al. used regression to evaluate the clinical factors that most influence the LOS in adult patients with peritonsillar abscesses [19]. Furthermore, Trunfio et al. used regression to predict the LOS of patients with femoral neck fractures at the University Hospital of Salerno [20].

Compared to other approaches for the analysis, simulation and modelling of biomedical data [21,22,23,24,25,26,27,28,29,30,31,32,33], machine learning (ML) techniques are useful in this context to the creation of a model that can help clinicians to manage patients; indeed, ML has been used in the literature for many aims. Regarding the diagnosis, it has been employed for Parkinson’s disease in neurology, in cardiology for the detection of coronary artery disease and in oncology for the classification of the tumour grade and the nodal status in oropharyngeal and oral cavity squamous-cell carcinoma [34]. Regarding the prognostic use of ML, it has allowed for the assessment of the risk of cardiac death or cardiovascular risks in several studies [35,36,37,38,39,40,41,42,43].

While in the abovementioned fields of medicine, ML has been widely applied for solving biomedical problems, and even outperforming human specialists in some cases, the review of Cabitza et al. outlines a different scenario in the orthopaedic field, where ML is still in a preliminary phase [44]. The authors compare ML to a health technology by stating that it needs assessments and evaluations in the real world setting in order to go from a Phase 2 trial, where it is now in orthopaedics, to a Phase 3 trial. In another study, Ramkumar et al. created an ML laboratory that was focused exclusively on orthopaedic surgery, with a two-fold aim: patient-specific value-based care, and human movement [45]. Moreover, interest in the use of ML to develop a predictive model of the hospital LOS has grown in recent years [46,47]. Researchers used a database composed of more than 120,000 patients to predict the LOS, measured in days, and the costs for patients who underwent a total hip and knee arthroplasty [48,49]. Similarly, Karnuta et al. developed a naïve Bayes ML algorithm and artificial neural networks to predict the LOS and costs for patients with fractures of the hip that used 103,592 patients [48,50,51]. More recently, Dogu et al. [47,52,53] integrated statistically based fuzzy cognitive maps and artificial neural networks to build an LOS prediction model for patients with an acute exacerbation of chronic obstructive pulmonary disease.

**Figure 1:**
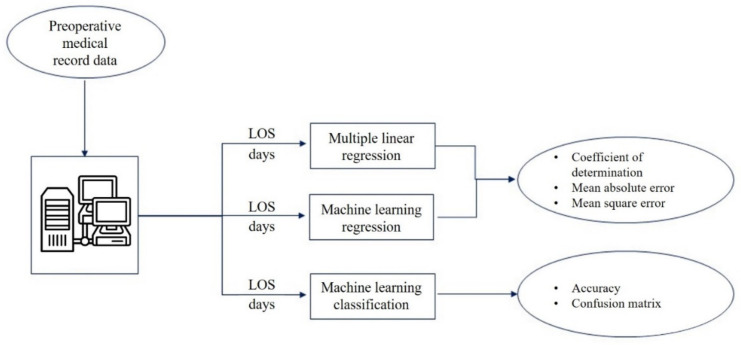
Workflow of the study.

The present study aims to propose a multiparametric approach that is based on both ML and multiple linear regression for LOS prediction. In this work, the preoperative data of elderly patients who were undergoing femur fracture surgery at the A. Cardarelli Hospital of Naples were collected, before and after the implementation of a DTAP, which was specifically dedicated to the management of elderly patients who had been diagnosed with fractures of the neck of the femur. All the gathered data were then used to model and predict the overall hospital LOS by following a three-way approach (see Figure 1): (i) A traditional multiple linear regression analysis; (ii) ML algorithms, which were trained and tested; and the obtained results were compared with (iii) An ML classification analysis, which was performed to predict the LOS grouped into weeks. The final aim of the study was not only to test and compare different prediction models that could support the estimation of the LOS starting from preoperative information, as already proposed in previous studies [48,49,50], but also to offer a general approach to assessing the impact of a newly implemented DTAP on the patients’ overall LOS.

## 2. Materials and Methods

### 2.1. Dataset

Data related to 1082 patients who underwent surgery due to a femur fracture were collected. They were extrapolated from the hospital′s information system (QUANI, which is owned by BIM Italia) and included patients who underwent surgery 14 months before and 14 months after the introduction of the DTAP, as also described in [54,55].

The following inclusion criteria were applied:

- Over 65 years of age;
- Primary or secondary discharge diagnosis: femur fracture.

The exclusion criteria were as follows:

- Polytrauma;
- Cancer as a primary or secondary diagnosis;
- Voluntary discharge;
- Death.

These criteria were taken from both the Essential Level of Assistance grid, released by the Italian Ministry of Health, and the hospital operative protocol.

The following variables were extracted and considered for subsequent analyses:

- Demographic information:
  ∘ Age.
- Timing information:
  ∘ Date and time of admission;
  ∘ Date and time of surgery;
  ∘ Date and time of discharge.
- Admission modality:
  ∘ Standard hospitalization;
  ∘ Hospitalization through the dedicated DTAP for femur fractures.
- Comorbid conditions:
  ∘ None;
  ∘ Allergies;
  ∘ Diabetes;
  ∘ Cardiovascular disease.
- Risk variables:
  ∘ American Society of Anesthesiologists (ASA) score.

In order to understand the influence of the variables considered for the LOS, a univariate statistical analysis was performed. First, a Kolmogorov–Smirnov test was performed on the data; then, since the *p*-values of the tests were lower than 0.05, the data were considered nonnormally distributed and a Mann–Whitney test was used to compare the mean LOS of the independent dichotomous variables (allergies, cardiovascular disease, diabetes, ASA score and DTAP), while a Kruskal–Wallis test was used to compare the mean LOS of the non-dichotomous variable (age). A significance level of 0.05 was adopted for the statistical analysis.

The group of researchers who collected and analysed the data was composed of the following:

- Biomedical engineers;
- An expert clinician in health management;
- The directors of the two departments of orthopaedics at the A. Cardarelli Hospital;
- The former director of the Complex Operative Unity of Health Planning and Programming at the A. Cardarelli Hospital;
- The Chief Medical Officer of the A. Cardarelli Hospital.

Table 1 shows the descriptive statistics of the dataset.

Table 1 shows that the most influent variables are the presence of the DTAP, cardiovascular diseases and a high ASA score, thus confirming the results obtained by previous studies in the literature [54,55]: its presence definitely reduces the preoperative LOS. 

### 2.2. The Diagnostic–Therapeutic–Assistance Path

International guidelines state that surgery within 48 h of admission is an effective treatment for significantly reducing short-, medium- and long-term complications [56]. In the A. Cardarelli Hospital, there was a nine-day average preoperative hospitalization, while the national average was four days. The proportion of hospitalizations for the fracture of the neck of the femur with surgery within 48 h in patients over 65 years of age was approximately 4%, versus 33% of the national average [57,58].

A. Cardarelli is not a single building, but a set of “pavilions”. Specifically, it consists of 21 pavilions, of which 14 are used for diagnosis and treatment, and the remaining 7 are used for technical and administrative services. Therefore, it was necessary to transport the patients among all the pavilions by ambulances in order to carry out the instrumental exams and visits. This was one of the major problems that arose during preoperative hospitalization and it suggests the need for a DTAP.

A DTAP was implemented to improve this situation at the A. Cardarelli Hospital; the plan consisted of 3 phases:

1. The early hospital assistance phase, which includes all preoperative exams and transfer to the orthopaedic pavilion;
2. The phase of perioperative management, which includes the anaesthesiologist evaluation, antibiotic prophylaxis and the acquisition of informed consent to be ready for surgery;
3. The postoperative phase and the predischarge period, which includes a rehabilitative treatment conducted by a multidisciplinary team (surgeon, physiotherapist, nurses and social worker).

Patients are discharged only after the Individual Rehabilitative Project has been sent to the directors of the districts where the patients live in order to guarantee that the necessary activities for the continuity of care are conducted according to their individual needs.

### 2.3. Multiple Linear Regression Model

Multiple linear regression (MLR) is a statistical technique that is used to investigate the relationship between more than two variables [20,59]. In particular, it is very useful in predicting the best relationship between a dependent variable and several independent variables [60]. This is the reason why, in this study, multiple linear regression was implemented with consideration to 7 independent variables, and the LOS as a dependent variable, and the following equation was obtained:

(1) $$y=\beta_{0}+\beta_{1}x_{1}+\beta_{2}x_{2}+\beta_{3}x_{3}+\beta_{4}x_{4}+{\;\beta }_{5}x_{5}+\beta_{6}x_{6}+\beta_{7}x_{7}$$

where y represents the LOS; $x_{i}$ is the considered variables; $\beta_{0}$ is the intercept; and $\beta_{i}$ is the regression coefficients. This equation enables the prediction of the LOS from the patient′s characteristics and further makes it possible to determine which of these characteristics most influences the output.

In order to use this model, however, the following assumptions must be verified:

1. That the relationship between the independent and dependent variables is linear;
2. That there is no multicollinearity in the data;
3. That the values of the residuals are independent;
4. That the variance in the residuals is constant;
5. That the values of the residuals are normally distributed;
6. That there are no influential cases biasing the model.

The analyses that were carried out with SPSS (Statistical Package for the Social Sciences) statistics software indicate that, in this case, the assumptions are verified, and the regression analysis could then be carried out. A significance level of 0.05 was adopted for the statistical and regression analysis. For more details on all the tests performed, please refer to the additional material that is provided with this paper (see Supplementary Materials from Tables S1–S8 and Figures S1–S17).

### 2.4. Machine Learning Analysis

In order to perform the ML analyses, the KNIME analytics platform was used, which is a business intelligence, or predictive analytical, tool that has already been employed in previous biomedical studies [61]. Both the regression and the classification analyses were performed with random forests (RFs), radial basis function (RBF) networks, multilayer perceptrons (MLPs) and support vector machines (SVMs). The first is an extension of the decision tree methodology through ensemble learning techniques, which are, namely, bagging and randomization: many trees are learned in parallel on a random subset of features, and the final prediction is produced by majority voting [62]. RBF and MLP are two examples of artificial neural network approaches [63,64], and they are thus characterized by an input layer, an output layer and hidden layers. Their differences are present in the literature: the basic difference is that the parameters of MLP are nonlinear, while those of RBF are linear. Finally, the SVM is an instance-based algorithm that assigns the class to the test data on the basis of their distance from similar training data. SVM is capable of addressing problems such as overfitting, small datasets and nonlinear and/or high-dimensional data; it can be used for both classification and regression [65,66,67].

These four ML algorithms were chosen because they are based on different operating principles: RF employs ensemble learning techniques on one of the most famous and applied ML algorithms (decision tree); SVM is clearly different from the previous algorithm since it is an instance-based algorithm, similar to the k nearest neighbours; while RBF and MLP are types of neural networks and are, thus, completely different from RF and SVM. Covering a different range of operating principles for the algorithms ensures an investigation of the data without bias caused by a single chosen algorithm. However, all the algorithms have been used in the literature for several biomedical studies, which shows their feasibility [68,69,70,71].

Since there were enough records in the dataset, the data were divided into training and test sets for the hold-out cross-validation, and the evaluation metrics were computed for both the regression and classification analyses. The coefficient of determination (R^2^), the mean absolute error and the root mean square error are used to evaluate the regression analysis, while, because of the high number of classes, the classification analysis is assessed with accuracy and presents the full confusion matrix.

## 3. Results

The dataset was divided into a training set and a test set (70% and 30% of the total, respectively) for the hold-out cross-validation. Then, a multiple linear regression analysis, RF, MLP, an RBF network and SVM were performed. The evaluation metrics on the test set are reported in Table 2 (multiple R^2^ are reported for all the tested models).

The regression coefficients and the *t*-test for the multiple linear regression are shown in Table 3.

The obtained predictive model can be considered valid, although the coefficient of determination (R^2^ = 0.610) is not very high. The ML algorithms obtained sufficient but not excellent results with regard to the coefficient of determination. The best one was obtained through the implementation of SVM (R^2^ = 0.617), but the RBF network obtained similar scoring (R^2^ = 0.616). The mean absolute error was similar for all the algorithms: it ranged between 2.000 (obtained by SVM) and 2.109 (obtained by MLP) days.

A second ML analysis was conducted with the same algorithms, but by performing a classification of the LOS, measured in weeks. The dataset was divided again into training and test sets with the same proportions previously mentioned, and the results are shown in Table 4.

The numbers reported in the confusion matrix represent the numbers of weeks. This confusion matrix is presented in order to show how the patient LOS, measured in weeks, is predicted by the best algorithm, and what the wrong predictions are (for example, 12 times, a patient with a one-week LOS received the prediction of a two-week LOS).

All the algorithms exceeded an 80% accuracy, and the most accurate was the RBF network, at 83.5%. The confusion matrix shows that the model was able to correctly identify the number of weeks for the LOS.

**Figure 2:**
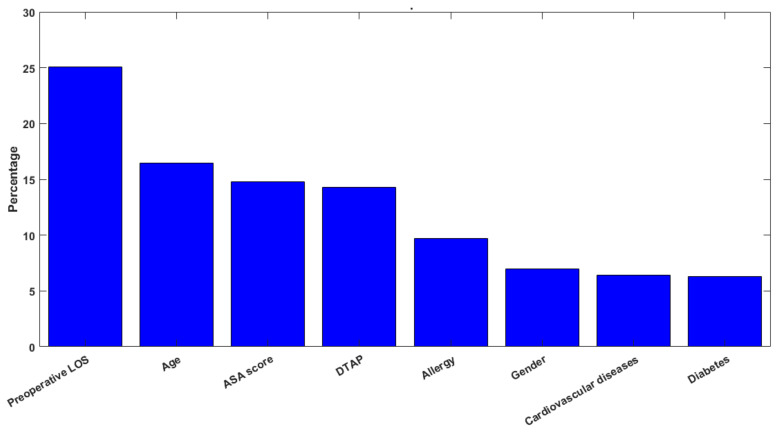
Histogram describing the feature importance.

Figure 2 shows the feature importance of the classification analysis computed on the RF that is based on the ratio between the number of splits performed through the considered feature, and the number of candidates for each level.

The most important feature was, of course, the preoperative LOS (25.1%), followed by the age (16.4%), the ASA score (14.8%) and the DTAP (14.3%). A comparison between the significance of the regression coefficients and the feature-importance ranking obtained by applying the machine learning models reveals that the most influencing factors, according to the RF algorithm, are the preoperative LOS and the age, which only partially overlaps the significance of the regression coefficients. Indeed, the preoperative LOS showed itself to be a significant predictor in both the multiple regression and machine learning models, while cardiovascular diseases assumed a higher significance as a predictor of the LOS in the regression analysis, rather than in the machine learning models. Such results suggest that the interpretation of the predictive models of the healthcare process should be carried out cautiously and in view of the value and effect of the chosen predictors used in the models. Indeed, the comparison of the predictors’ relevance in the examined regression and classification models is an essential part in the assessment of the validity of the findings, and it should be the guidance to achieving reasonable and interpretable results when dealing with predictive algorithms in the healthcare context.

## 4. Discussion and Conclusions

In this paper, multiple linear regression and several ML algorithms were used to provide a model that is able to predict the LOS (measured in days) of patients hospitalized for femur fracture at the A. Cardarelli Hospital of Naples. Moreover, ML algorithms were applied to classify the LOS, grouped by weeks. In addition, the analyses have provided indications of the variables that most influence the LOS, and they agree with the results that have been obtained in the literature with regard to the application of DTAPs [54,55]. The feature importance, which is represented in Figure 2, also confirms what the literature has expressed over the years. First, reducing the preoperative LOS has a preeminent role in the overall hospital stay because, as proven by previous studies [4,5], patients undergoing surgery within 48 h from admission to the hospital experience lower complications and, consequently, a reduced LOS. Furthermore, despite being in fourth place in feature importance, the presence of the DTAP on patients who are affected by a fracture of the femur provides the classification of the LOS with almost the same contribution from the age and the ASA score. Indeed, this is another confirmation of the literature because Improta et al. and Ricciardi et al. show that the use of a DTAP was useful to decrease the preoperative and overall LOS [54,55].

The errors of the classification ML analysis were mostly just one week. The misclassified records were those patients with the highest number of weeks for the LOS because the number of cases in the dataset was not large enough to train and test the models.

Although this paper focuses only on the LOS, it holds technical value as it applies a wide range of ML algorithms that have not been previously investigated, such as RF, the RBF network, MLP and SVM, since previous researchers have employed only a naïve Bayes model [48,49,50]. The study achieved good results, with accuracies beyond 80% in the classification of the LOS. The aim of this research was to improve the models and to change the perspective. In the first analysis, although it was a hard task, the regression performed punctual predictions since the LOS was measured in days, with discrete results (the R^2^ was greater than 0.60). In the second analysis, the LOS was grouped into four classes by week, and a harder prediction had to be performed (in previous research, it was grouped into “1–2 days”, “3–5 days” and “6+ days” [48,49]), but the results were good again.

A limitation of this work could be the presence of a clinical pathway (DTAP) that was specific to the hospital of Naples, which would not allow the models to be used in many other hospitals. Indeed, regarding the possibility of importing this path into other facilities, it must be mentioned that the distributed pavilion-based structure of the facility is an intrinsic characteristic of A. Cardarelli that cannot be found in all hospitals; therefore, the DTAP could be useful for hospitals that are organized and structured in similar ways. An example of an analogous pathway is reported in [72], where the authors discuss the implementation of a DTAP for patients with femur fracture in the Italian hospital, “San Giovanni di Dio e Ruggi d’Aragona”, of Salerno. Some similarities can be found between the two DTAPs, such as the presence of a protocol for the rapid transfer of the patient from the emergency department to the orthopaedic ward, the timely multiprofessional (orthopaedic, internal medicine, anaesthesiology and nursing) assessment and, finally, the early rehabilitation care.

Nevertheless, this clinical pathway has earned trust from health policy and it has also been discussed in other Italian regions. The collection of more variables, rather than more patients or the testing of different algorithms, is fundamental in order to obtain further improvements to these models. Although the models that were obtained are not perfectly accurate predictors of the LOS, the obtained results are promising, and since the selected predictors are readily available in the patients’ clinical records, and since the adopted algorithms do not require time-consuming procedures or dedicated hardware, the proposed methodology may be applicable in other settings, and may potentially represent a support tool for the management of department resources and workflows.

## Figures and Tables

**Table 1:** Descriptive statistics of the dataset.

Variables	Categories	*N*	LOS (Days) [Average ± SD]	*p*-Value
Age (years)	<75	225	11.46 ± 5.527	0.399
	75–90	720	11.21 ± 5.071	
	>90	137	10.79 ± 4.538	
Allergies	Yes	138	11.79 ± 5.609	0.153
	No	944	11.12 ± 5.025	
Cardiovasculardiseases	Yes	897	11.36 ± 4.987	0.002
	No	185	10.48 ± 5.601	
Diabetes	Yes	268	11.56 ± 5.657	0.291
	No	814	11.40 ± 5.162	
ASA score	I-II	94	9.21 ± 3.970	<0.001
	III-IV	988	11.39 ± 5.242	
DTAP	No	534	13.21 ± 5.126	<0.001
	Yes	548	9.25 ± 4.259	

**Table 2:** Evaluation metrics for the regression analysis of LOS, measured in days.

	Multiple Linear Regression	Random Forests	MLP	RBF Network	SVM
R^2^	0.610	0.507	0.584	0.616	0.610
Mean absolute error	3.987	2.45	2.109	2.077	2.000
Mean squared error	11.624	11.949	10.075	9.302	9.268

**Table 3:** Coefficients of the multiple linear regression model.

Variables	Regression $\mathrm{Coefficients}\;(\beta_{i})$	t	*p*-Value
Intercept	3.42	2.24	0.02
Age	−0.01	−0.85	0.39
ASA score	1.22	−0.76	0.45
Diabetes	−0.21	−0.75	0.45
Cardiovascular diseases	−0.25	3.83	0.001
Allergies	0.04	0.11	0.91
Preoperative LOS	1.03	27.77	<0.001
DTAP	0.35	1.23	0.22

**Table 4:** The accuracies and the best confusion matrix for the classification analysis of LOS, measured in weeks.

	RF	MLP	RBF Network	SVM
Accuracy (%)	81.1	82.3	83.5	81.1
The best confusion matrix (RBF network)				
Real/Predicted	1	2	3	4
1	154	12	0	0
2	29	108	1	0
3	1	5	17	0
4	1	2	4	0

## Data Availability

The data cannot be made available due to privacy restrictions.

## Supplementary Materials

The following supporting information can be downloaded at: https://www.mdpi.com/article/10.3390/bioengineering9040172/s1: Tables S1–S8 and Figures S1–S17, which report the methodological details of the multiple linear regression model, are available as Supplementary Materials.
