# Supplementary material for: Machine Learning and Regression Analysis to Model the Length of Hospital Stay in Patients with Femur Fracture
Source: Bioengineering (Basel). 2022 Apr 14;9(4):172. doi: 10.3390/bioengineering9040172 (PMC9029792; doi:10.3390/bioengineering9040172)
Supplement: Supplementary file 1 [file bioengineering-09-00172-s001.zip › bioengineering-1621207-supplementary.pdf]

bioengineering

MDPI

Supplementary Materials

Machine Learning and Regression Analysis to Model the Length of Hospital Stay in Patients with Femur Fracture

Carlo Ricciardi [1,†] Alfonso Maria Ponsiglione [1,*,†], Arianna Scala [2], Anna Borrelli [3], Mario Misasi [4], Gaetano Romano [4], Giuseppe Russo [5], Maria Triassi [2,6] and Giovanni Improta [2,6]

[1] Department of Electrical Engineering and Information Technology, University of Naples "Federico II", Naples, 80125, Italy; carlo.ricciardi@unina.it
[2] Department of Public Health, University Hospital of Naples "Federico II", 80131 Naples, Italy; ariannascala7@gmail.com (A.S.); triassi@unina.it (M.T.); ing.improta@gmail.com (G.I.)
[3] Health Department, University Hospital of Salerno "San Giovanni di Dio e Ruggi d'Aragona", Salerno, 84126, Italy; acquarama@libero.it
[4] National Hospital (A.O.R.N.) Antonio Cardarelli, Department of the Orthopaedics, Naples, 80131, Italy; mario.misasi@aocardarelli.it (M.M.); gaetano.romano@aocardarelli.it (G.R.)
[5] National Hospital (A.O.R.N.) Antonio Cardarelli, Naples, 80131, Italy; ariete_gr@libero.it
[6] Interdepartmental Center for Research in Healthcare, Management and Innovation in Healthcare (CIRMIS), University of Study of Naples "Federico II", Naples, 80131, Italy
* Correspondence: alfonsomaria.ponsiglione@unina.it
† These authors equally contributed to the work.

1. Multiple Regression and Statistical Tests

In this work the tests on the available dataset were performed with the SPSS (Statistical Package for Social Science) software in order to verify the assumptions of the multiple linear regression model. In this supplementary file, the results obtained for each of the 6 assumptions are reported.

1.1. The Relationship between the Independent and Dependent Variable is Linear

This assumption must be verified for all variables: age, cardiovascular disease, diabetes, American Society of Anaesthesiologists (ASA) score, allergies, pre-operative LOS and DTAP. For each of them a scatter plot where, on the x-axis, there is the independent variable and, on the y-axis, the dependent variable (LOS) is reported. Then the partial regression plots where, on the x-axis, there are the residues of the independent and, on the y-axis, there are the residues of LOS are shown. In addition, for dichotomous and categorical variables, the correlation with LOS was calculated using also Point Biserial Correlation. Figures S1 and S2 show that the assumption of linearity is satisfied for the variable "age".

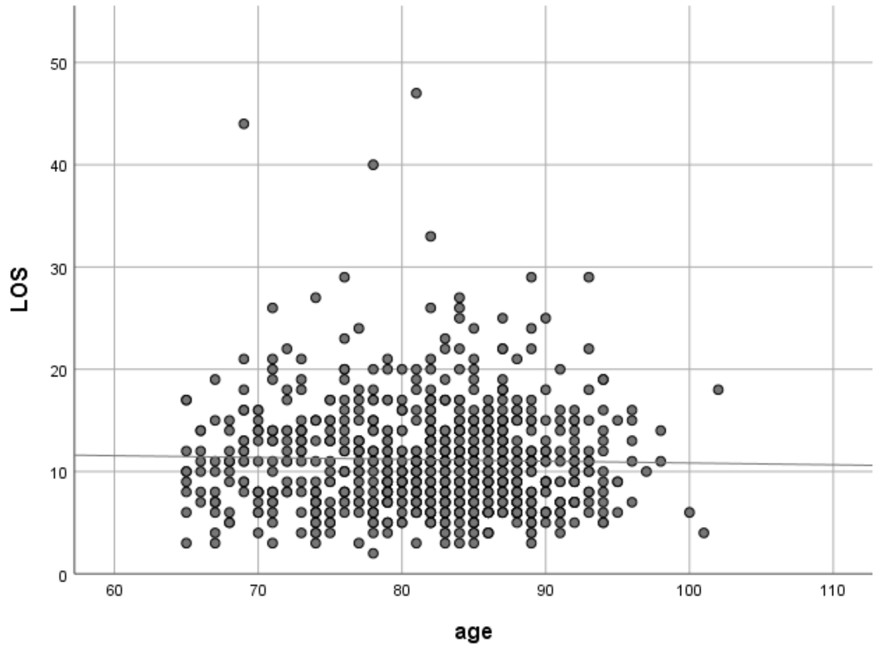

Figure S1. Partial Regession Plot (age).

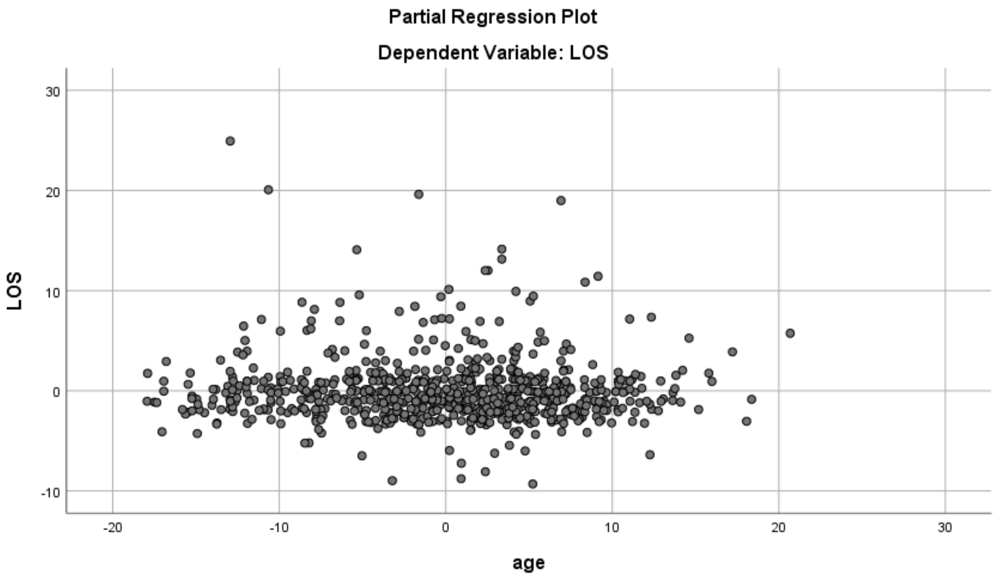

Figure S2. Partial Regession Plot (age).

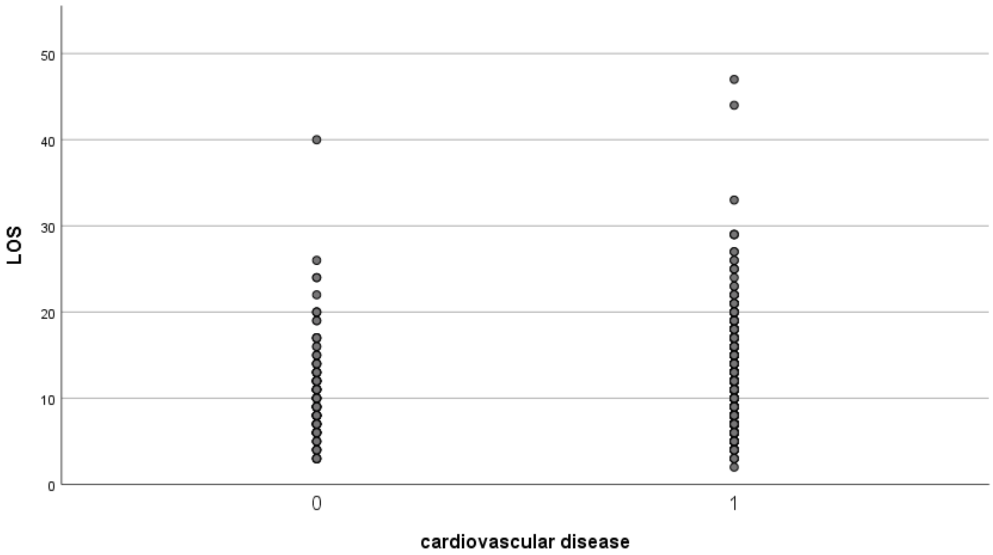

Figure S3. Scatter Plot (cardiovascular disease).

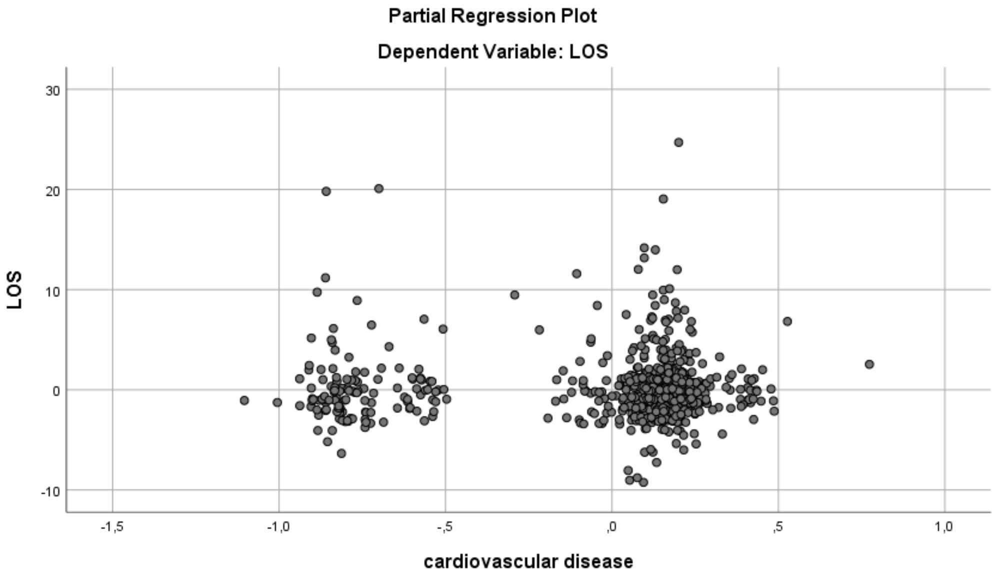

Figure S4. Partial Regression Plot (cardiovascular disease).

Table S1. Point Biserial Correlation (cardiovascular disease).

		**cardiovascular disease**	**LOS**
**cardiovascular disease**	*Point-biserial correlation coefficient*	1	0.072
	p-value (2-tailed)		0.048
	N	757	757
**LOS**	*Point-biserial correlation coefficient*	0.072*	1
	p-value (2-tailed)	0.048	
	N	757	757

Since "cardiovascular disease" is a dichotomous variable, in addition to the scatter plots (Figures S3 and S4), the point biserial correlation coefficient was also calculated. The test is slightly statistically significant with a p-value of 0.048 on a significance level of 0.05 (Table S1), so we can conclude that there is a slightly positive correlation, which is statistically significant between cardiovascular disease and the LOS.

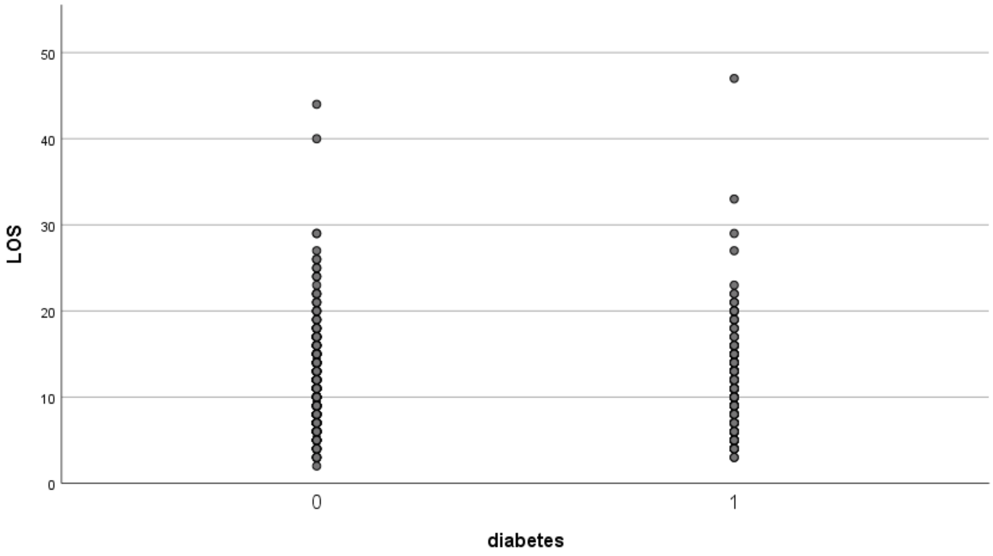

Figure S5. Scatter Plot (diabetes).

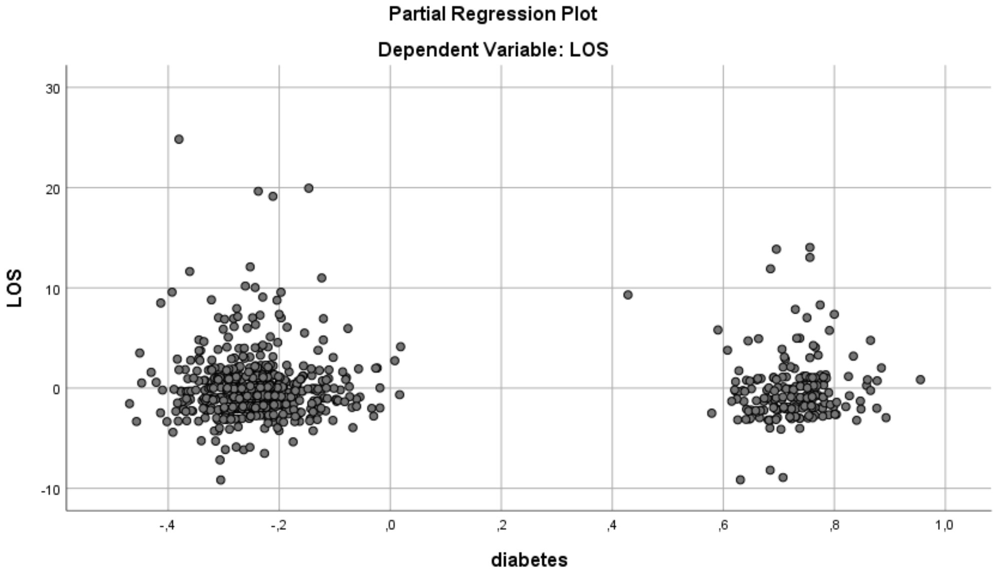

Figure S6. Partial Regression Plot (diabetes).

Table S2. Point Biserial Correlation (diabetes).

		LOS	diabetes
**LOS**	*Point-biserial correlation coefficient*	1	0.049
	p-value (2-tailed)		0.176
	N	757	757
**diabetes**	*Point-biserial correlation coefficient*	0.049	1
	p-value (2-tailed)	0.176	

N	757	757

In this case the point biserial correlation coefficient returns a value of about 0.05 with a p-value of 0.176 (Table S2). This allows us to state that the variable "diabetes" (Figures S5 and S6) shows a non-significant correlation with the LOS.

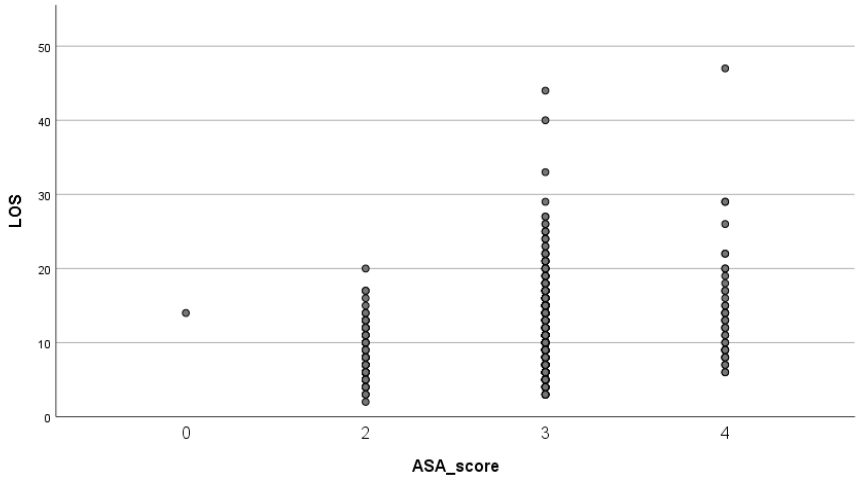

Figure S7. Scatter Plot (ASA score).

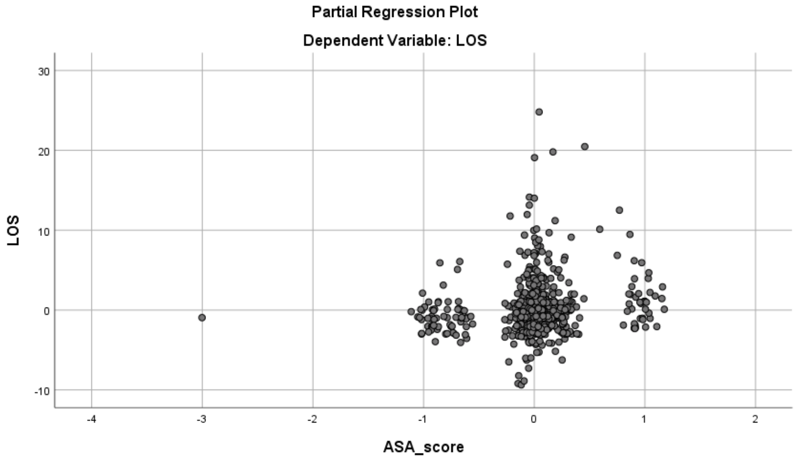

Figure S8. Partial Regression Plot (ASA score).

Table S3. Point Biserial Correlation (ASA score).

		ASA_score	LOS
**ASA_score**	*Point-biserial correlation coefficient*	1	0.188
	p-value (2-tailed)		0.000
	N	757	757
**LOS**	*Point-biserial correlation coefficient*	0.188	1
	p-value (2-tailed)	0.000	
	N	757	757

The test is statistically significant for the categorical variable "ASA score" (Table S3). A slightly positive correlation with the dependent variable LOS is found (Figures S7 and S8).

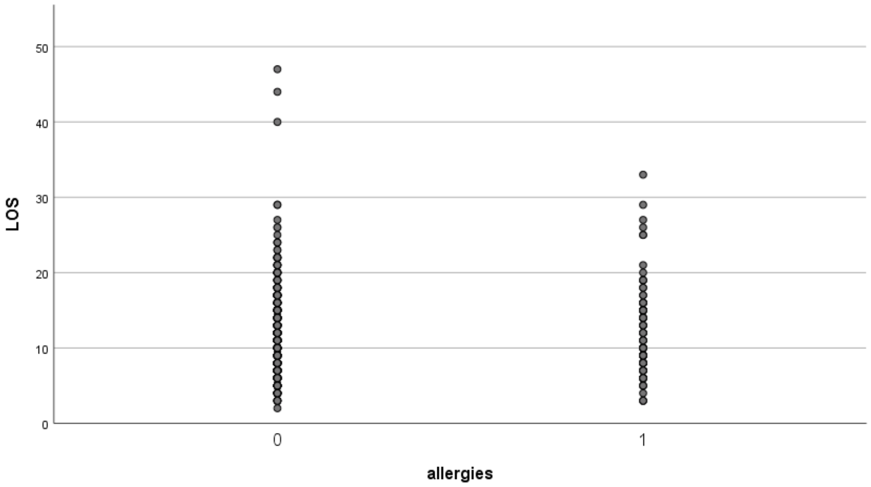

Figure S9. Scatter Plot (allergies).

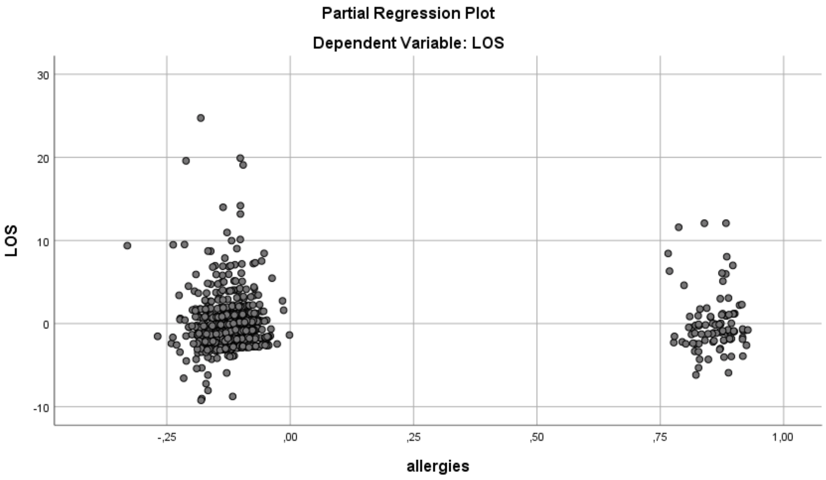

Figure S10. Partial Regression Plot (allergies).

Table S4. Point Biserial Correlation (allergies).

		LOS	allergies
**LOS**	*Point-biserial correlation coefficientn*	1	0.047
	p-value (2-tailed)		0.199
	N	757	757
**allergies**	*Point-biserial correlation coefficient*	0.047	1
	p-value (2-tailed)	0.199	
	N	757	757

For the variable "allergies" (Figures S9 and S10), there is a slightly positive correlation with the LOS, which however is not statistically significant. In fact, from Table S4 it is possible to notice that the results of the test are not significant (p-value > 0.05).

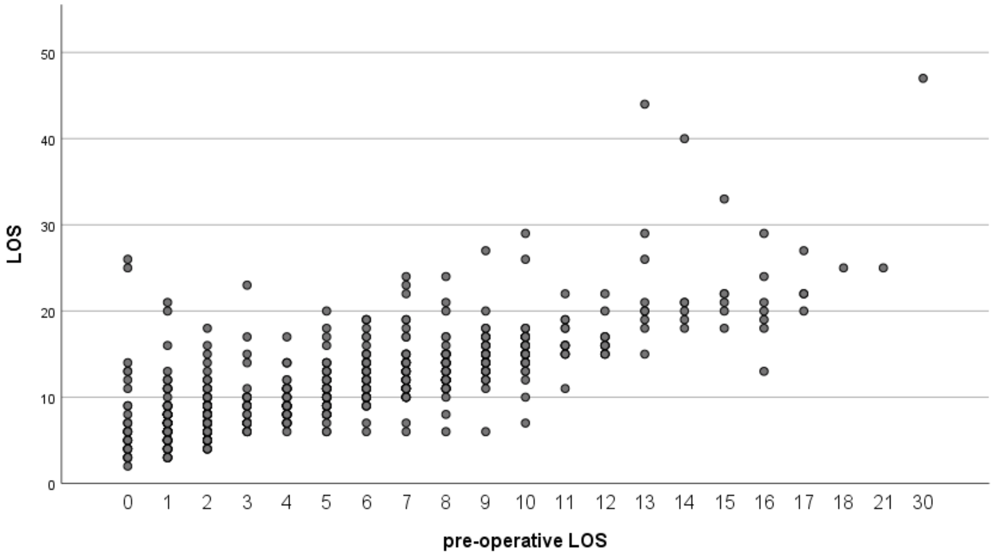

Figure S11. Scatter Plot (pre-operative LOS).

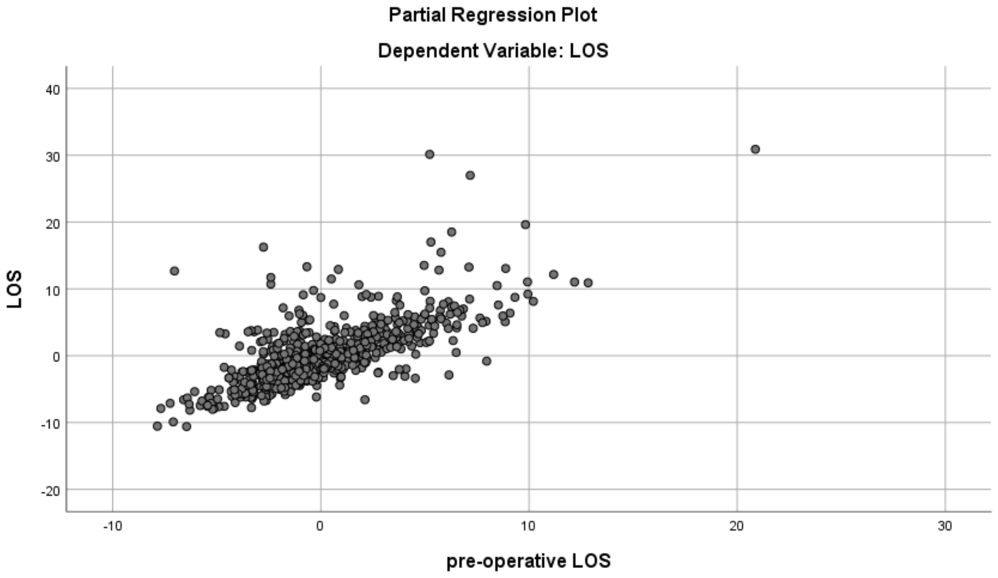

Figure S12. Partial Regression Plot (pre-operative LOS).

The linearity between LOS and pre-operative LOS is evident (Figures S11 and S12). The partial regression plot also indicates that the independent variable is very useful for regression analysis. This was predictable since LOS is the sum of pre-operative and post-operative LOS.

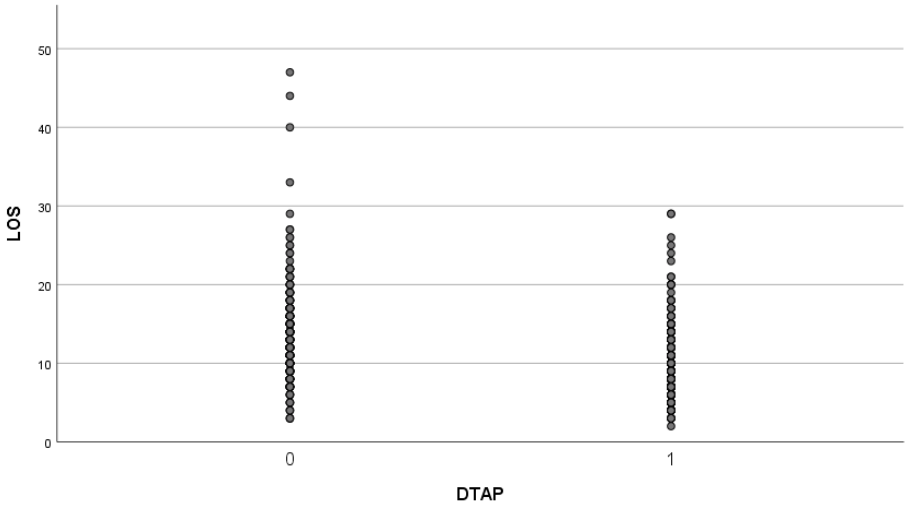

Figure S13. Scatter Plot (DTAP).

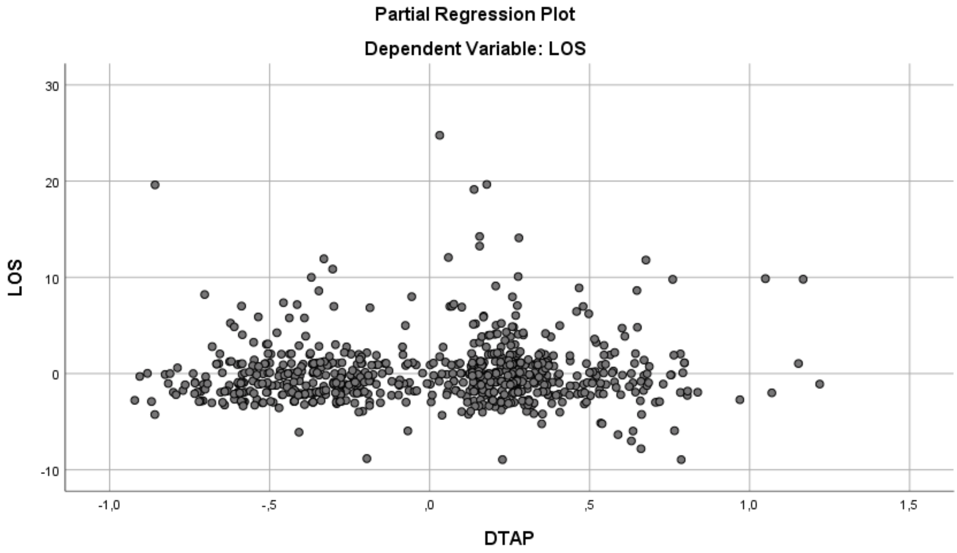

Figure S14. Partial Regression Plot (DTAP).

Table S5. Point Biserial Correlation (DTAP).

		LOS	DTAP
**LOS**	*Point-biserial correlation coefficient*	1	-0.398
	p-value (2-tailed)		0.000
	N	757	757
**DTAP**	*Point-biserial correlation coefficient*	-0.398**	1
	p-value (2-tailed)	0.000	
	N	757	757

Finally, for the variable "DTAP" (Figures S13 and S14), there is a negative correlation with the LOS, which is statistically significant (Table S5).

1.2. There is no Multicollinearity in the Data

All variables must be independent of each other. In order to verify this assumption, it is necessary to compute the correlation matrix. The correlation analysis produces a sample correlation coefficient which measures the strength and direction of linear relationships between pairs of continuous variables.

Table S6. Pearson's Bivariate Correlation test.

		LOS	age	cardiovascular disease	diabetes	ASA_score	allergies	pre-operative LOS	DTAP
**Correlation coefficient**	LOS	1.000	-0.025	0.072	0.049	0.188	0.047	0.774	-0.398
	age	-0.025	1.000	0.141	-0.052	0.177	-0.044	-0.025	-0.010
	cardiovascular disease	0.072	0.141	1.000	0.114	0.253	-0.031	0.091	0.001
	diabetes	0.049	-0.052	0.114	1.000	0.109	-0.007	0.073	0.008
	ASA_score	0.188	0.177	0.253	0.109	1.000	0.036	0.134	0.003
	allergies	0.047	-0.044	-0.031	-0.007	0.036	1.000	0.048	0.047
	pre-operative LOS	0.774	-0.025	0.091	0.073	0.134	0.048	1.000	-0.553
	DTAP	-0.398	-0.010	0.001	0.008	0.003	0.047	-0.553	1.000

Table S7. Coefficients and collinearity statistics of the multiple linear regression model.

	Unstandardized Coefficients		Standardized Coefficients	t	p-value	Collinearity Statistics	
	B	Std. Error	Beta			Tolerance	VIF
**intercept**	3.422	1.525		2.244	0.025		
**age**	-0.014	0.017	-0.020	-0.853	0.394	0.946	1.057
**cardiovascular disease**	-0.252	0.332	-0.018	-0.762	0.447	0.911	1.098
**diabetes**	-0.208	0.278	-0.017	-0.747	0.455	0.969	1.032
**ASA_score**	1.227	0.320	0.093	3.831	0.000	0.887	1.127
**allergies**	0.039	0.361	0.003	0.109	0.913	0.985	1.015
**pre-operative LOS**	1.030	0.037	0.782	27.770	0.000	0.661	1.514
**DTAP**	0.353	0.288	0.034	1.226	0.221	0.679	1.473

The assumption turns out to be satisfied. In fact, the correlation coefficients always assume values lower than 0.8 (Table S6) and the collinearity statistics (Table S7) always show a tolerance higher than 0.5 and VIF lower than 2.

1.3. The Values of the Residuals are Independent

In order to verify this assumption, it was necessary to perform a Durbin-Watson test. The Durbin Watson Test is a measure of autocorrelation (also called serial correlation) in residuals from regression analysis. Autocorrelation is the similarity of a time series over successive time intervals. It can lead to underestimates of the standard error and can cause you to think predictors are significant when they are not.

Table S8. Durbin-Watson test.

Value of the Durbin-Watson test
2.029

The value of the Durbin-Watson test between 1 and 3 (Table S8) so the values of residuals are independent.

1.4. The Variance of the Residuals is Constant

It was necessary to test the homoscedasticity of the data with a scatter plot with "standardised expected value regression" on the x-axis and "standardised residual regression" on the y-axis (Figure S15).

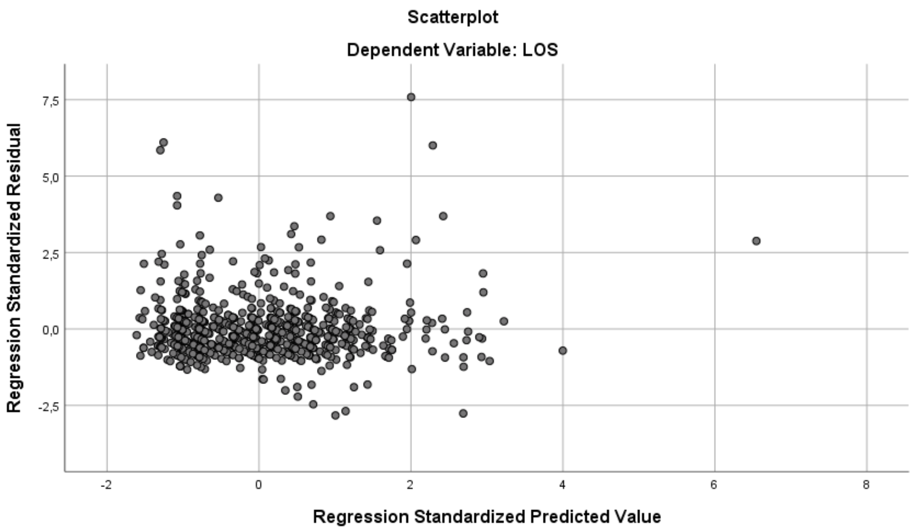

Figure S15. Homoscedasticity of the data.

The scatter plot does not show a clear correlation between the residues and the expected values.

1.5. The Values of the Residuals are Normally Distributed

This was tested with a P-P graph (Probability-Probability graph). This graph allows to assess how similar two sets of data are, by plotting the two cumulative distribution functions on a graph (CDF).

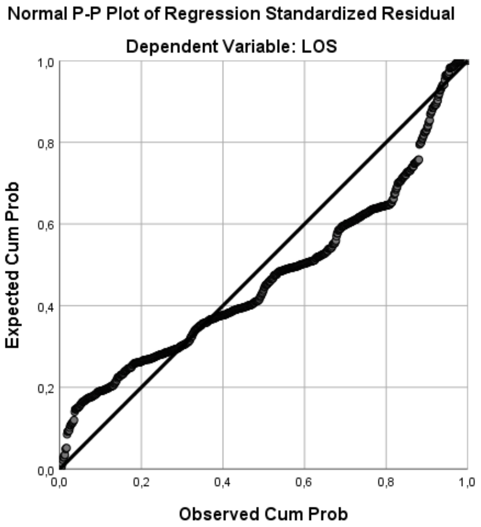

Figure S16. P-P graph.

The points almost all deviate from the diagonal line, although not by much (Figure S16).

1.6. There are no Influential Cases Biasing the Model

It was necessary to check that all Cook's Distance values are less than 1. Cook's distance is used in Regression Analysis to find influential outliers in a set of predictor variables. In other words, it is a way to identify points that negatively affect your regression model. The measurement is a combination of each observation's leverage and residual values; the higher the leverage and residuals, the higher the Cook's distance.

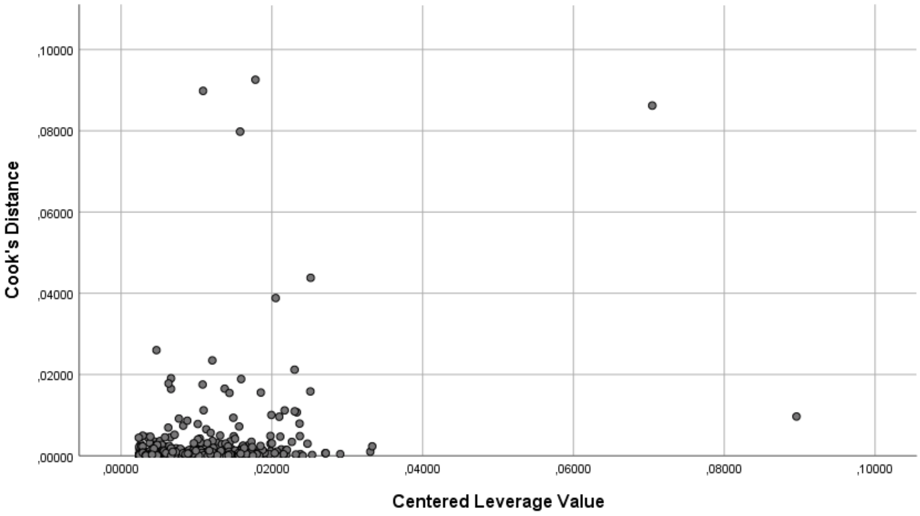

Figure S17. Cook's Distance.

As it can be seen in the graph (Figure S17) all the Cook's Distance values are well below 1.
